# Molecular aspects of COVID-19 and its relationship with obesity and physical activity: a narrative review

**DOI:** 10.1590/1516-3180.2021.1038.R1.06072022

**Published:** 2022-09-12

**Authors:** Ramon de Souza Lino, Mariana Sousa de Pina Silva, Daniel Simões de Jesus, Rodrigo Colares de Macedo, Laura Souza Lagares, Felipe Nunes Almeida dos Santos, Luiz Alberto Bastos de Almeida, Eric Simas Bomfim, Clarcson Plácido Conceição dos Santos

**Affiliations:** I BSc. Physical Education Professional, Research Group on Metabolic Diseases, Physical Exercise and Health Technologies, Escola Bahiana de Medicina e Saúde Pública (EBMSP), Salvador (BA), Brazil.; II Undergraduate Student, Research Group on Metabolic Diseases, Physical Exercise, and Health Technologies, Escola Bahiana de Medicina e Saúde Pública (EBMSP), Salvador (BA), Brazil.; III PhD. Assistant Professor, William Harvey Research Institute, Barts and the London School of Medicine and Dentistry, Queen Mary University of London, London, United Kingdom.; IV Undergraduate Student, Research Group on Metabolic Diseases, Physical Exercise and Health Technologies, Escola Bahiana de Medicina e Saúde Pública (EBMSP), Salvador (BA), Brazil.; V BSc. Physical Education Professional, Research Group on Metabolic Diseases, Physical Exercise and Health Technologies, Escola Bahiana de Medicina e Saúde Pública (EBMSP), Salvador (BA), Brazil.; VI BSc. Physical Education Professional, Research Group on Metabolic Diseases, Physical Exercise and Health Technologies, Escola Bahiana de Medicina e Saúde Pública (EBMSP), Salvador (BA), Brazil.; VII MSc. Assistant Professor, Laboratory of Physical Activity, Universidade Estadual de Feira de Santana (UEFS), Feira de Santana (BA), Brazil.; VIII BSc. Physical Education Professional, Research Group on Metabolic Diseases, Physical Exercise and Health Technologies, Escola Bahiana de Medicina e Saúde Pública (EBMSP), Salvador (BA), Brazil;; Physical Education Professional, Department of Physical Education, Obesity Treatment and Surgery Center, Salvador (BA), Brazil; IX PhD. Assistant Professor, Research Group on Metabolic Diseases, Physical Exercise and Health Technologies, Escola Bahiana de Medicina e Saúde Pública (EBMSP), Salvador (BA), Brazil.

**Keywords:** Obesity, COVID-19, Inflammation, Metabolism, SARS-CoV-2, Body weight, Physical activity, Fatness, Severe acute respiratory syndrome coronavirus 2

## Abstract

**BACKGROUND:**

Severe acute respiratory syndrome coronavirus 2 has several mechanisms of action related to inflammatory responses, especially in individuals diagnosed with obesity. This hyperinflammatory clinical profile resulting from the association between obesity and coronavirus disease 2019 (COVID-19) may be attenuated by regular physical activity.

**OBJECTIVE:**

The aim of this study was to review the evidence on the consequences of physical inactivity and physical activity on COVID-19 in patients with obesity.

**DESIGN AND SETTING:**

Narrative review at the Bahiana School of Medicine and Public Health in Salvador, Brazil.

**METHODS:**

We searched evidence on the association of COVID-19 with physical activity and obesity using the following keywords: “covid-19,” “physical activity,” and “obesity”. The databases used were MEDLINE (PubMed), ScienceDirect, and Virtual Health Library. Studies published from 2019 to 2021 and available in Portuguese, English, and Spanish were included. The final search was conducted on September 26, 2021.

**RESULTS:**

We identified 661 studies in the database, among which 71 were considered for inclusion in the narrative review of the molecular aspects of COVID-19 and its relationship with physical activity and obesity.

**CONCLUSION:**

This literature review enabled the perception of the relationship between the molecular mechanisms of COVID-19 and obesity. Regular physical activity had various benefits for the inflammatory condition of the studied population, highlighting moderate-intensity.

## INTRODUCTION

December 2019 was marked by the first recorded case of severe acute respiratory syndrome coronavirus 2 (SARS-CoV-2; coronavirus disease 2019, COVID-19), which affects the respiratory system with a high viral load and has the potential to cause hospitalization and death.^1^ Since then, a global pandemic has been declared, affecting population health in two ways: directly, from contact with the virus and its pathological evolution in the infected individual, and indirectly, owing to the need to restrict human contact to reduce the risk of contagion, from social isolation.^2^ Research has been conducted to uncover the causes of infection, methods of transmission, and effective measures of prevention, taking into account at-risk groups that need specific attention; this highlighted the high risk for individuals diagnosed with obesity.^3^

Lockdowns, to reduce human–human contact and prevent the spread of SARS-CoV-2, cause social isolation and reduce physical activity (PA), increasing the number of physically inactive individuals; this increases their body fat percentage, making them part of the high-risk group for COVID-19.^4^ A recent cohort study with approximately 490 000 participants indicated that a body mass index above 30 kg/m^2^ is associated with a higher risk of developing a severe viral infection, requiring hospitalization, with an odds ratio of 1.73.^5^ This increased risk seems to be related to inflammatory mechanisms and is associated with a worse prognosis to COVID-19, highlighting the action of angiotensin-converting enzyme 2 (ACE2) and interleukin (IL)-6, which are expressed in adipose tissue and, respectively, allow the entry of SARS-CoV-2 and maintain the inflammatory state of individuals with obesity, especially those infected with the virus.^2,6^

Regular PA seems to attenuate weight gain and reduce the inflammation present in adults.^7^ A recent systematic review reported strong evidence on the relationship between PA for more than 150 min per week and the attenuation of weight gain in adults, corroborating the recent World Health Organization update on PA recommendations during the COVID-19 pandemic.^7,8^ PA seems to reduce the inflammatory state of individuals with obesity through molecular changes in the adipose tissue, attenuating the action of inflammatory mechanisms, based on the expression of proteins, such as peroxisome proliferator-activated receptor γ co-activator 1α (PGC-1α), that favor mitochondrial biogenesis, and activation of molecular pathways, such as the AMP-activated protein kinase (AMPK) pathway, that, given increased enzymatic activity, act directly on lipid metabolism.^9,10^

There are gaps in the literature regarding COVID-19 and its molecular mechanisms, to the detriment of the population diagnosed with obesity, relating to effective methods to alleviate the exacerbated responses to which these individuals are exposed, given the inflammatory mechanism of these two pathologies.

## OBJECTIVE

The aim of this study was to review the evidence regarding the consequences of physical inactivity and exercise on COVID-19 in patients with obesity.

## METHODS

This study was an integrative literature review. The databases used to search for articles were MEDLINE (PubMed), ScienceDirect, and Virtual Health Library. Articles published from 2019 to 2021, available in Portuguese, English, and Spanish, were included, independent of the study design. The final search was performed on September 26, 2021.

The search strategy used in PubMed involved synonyms of COVID-19, obesity, and PA identified in Medical Subject Headings and Descriptors in Health Sciences: (((((Exercises) OR (Physical Activity)) OR (Exercise Training))) AND (((Obesity) OR (Obesity, Abdominal)) OR (Overweight))) AND ((((COVID 19) OR (SARS-CoV-2 Infection)) OR (COVID-19 Pandemic)) OR (Coronavirus Disease-19)).

The search was performed on the informed database. Duplicate articles were removed and filtered, based on the inclusion criteria, using the following order: reading of the titles, abstracts, and full text. In addition to the search strategy, some articles were accessed manually from reference lists.

## RESULTS

**Figure 1.**
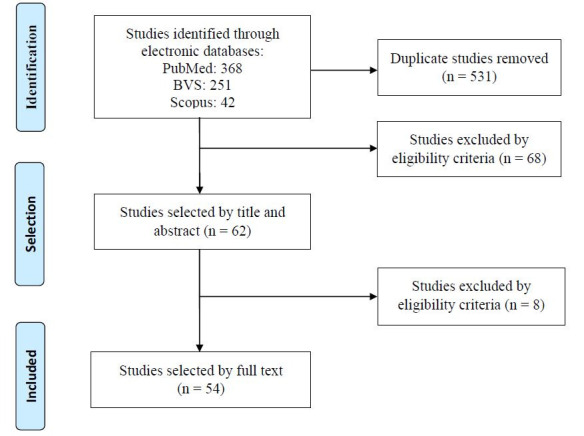
Flowchart of database searches and selection process.

The results of the search and selection strategy are shown in the flowchart (Figure 1).

### Inflammatory mechanisms of obesity

Obesity, a chronic disease associated with inflammatory responses, can develop owing to multiple factors, including genetic predisposition, emotional disorders, hormonal changes, and energy imbalance between high caloric intake and low caloric expenditure, resulting in the proliferation of excess adipose tissue.^11,12,13^ This highly pro-inflammation compromises several metabolic mechanisms, such as glycemic regulation and beta-oxidation, and causes endothelial and vascular dysfunctions.^14^ In general, the levels of several proteins and cytokines related to inflammatory responses are above the standards in obese individuals. A recent literature review identified elevated C-reactive protein levels (≥ 3 mg/L) in 14.4% of individuals with normal body mass index and 36% of obese individuals.^15^

According to Zeyda and Stulnig,^16^ the greater the amount of adipose tissue, the greater the secretion of interleukin (IL)-6, with visceral adipose tissue being the main factor responsible for the secretion of this cytokine, which has local and systemic mechanisms of action. In addition to visceral adipose tissue, IL-6 may originate in skeletal and smooth muscle tissue and endothelial, liver, and pancreatic cells.^14,17,18^ Among various possible outcomes, the release of IL-6 from these structures results in the increased production of triacylglycerol by the liver, inhibition of lipase and hepatocyte insulin receptors, and, consequently, insulin resistance.^16,19^ The positive correlation between IL-6 and C-reactive protein (CRP) levels results in an immunoregulatory function, as CRP is responsible for mediating the acute inflammatory response to aggressive agents and activating the complement system, which activates inflammatory processes and acts via the classical pathway, in which C proteins are linked to the target microorganism, exposing the activation sites of the system and subsequently generating the cleavage process of these components, ranging from C1 to C9, thereby allowing defense against aggressive agents, in parallel to antibodies.^20^ Elevated CRP levels are also associated with cardiovascular and metabolic pathologies, such as type 2 diabetes mellitus.^15,21,22^

Dai et al.^23^ have highlighted tumor necrosis factor-alpha (TNF-α) as a cytokine that also plays a crucial role in obesity. Although not primarily synthesized in adipocytes, the messenger ribonucleic acid for the production of TNF-α is present in adipose tissue, which is then used for TNF-α synthesis by macrophages. Thus, TNF-α plays an important role in adipose tissue, where the production of cytokines such as IL-6, cellular processes such as apoptosis, and vascular processes such as angiogenesis occur.^23^ It has been suggested that a high body mass index is associated with higher levels of TNF-α in the systemic circulation.^24,25,26^

Other important inflammatory markers are adipokines, cytokines secreted by adipose tissue, which affect several metabolic pathways, as their secretion results in an immune system response to the aggressor agent, releasing leukocytes in response to systemic inflammation. Thus, obesity, at the molecular level, may have a systemic inflammation origin and not only be caused by adipose tissue dysfunction.^27^ Adipokines are also directly linked to vascular homeostasis; the microcirculation present in the adipose tissue can be compromised by the growth and increase in the number of adipocytes, resulting in deficient local and systemic circulation. This creates the need for angiogenesis to avoid severe impairments, such as adipocyte necrosis and worsening of adipose tissue inflammation, induced by hypoxia in obese individuals.^28,29,30,31,32^

### Obesity and COVID-19

The mechanism of action of COVID-19 may be related to inflammatory immune responses caused by contact between the virus and pulmonary epithelium. Individuals with impaired immune responses, as well as those affected by chronic non-communicable diseases, have previous inflammatory conditions; thus, they are prone to complications caused by this infection, among whom individuals diagnosed with obesity are at greater risk.^33^

In a recent literature review, Stefan et al.^34^ identified that the risk of this infection worsening is greater in obese patients, especially in those with high levels of visceral fat. This occurs because visceral adipose tissue is one of the major factors responsible for the expression of inflammatory mediators related to obesity.^34^ Moreover, excess adipose tissue, especially visceral adipose tissue, is associated with the greater presence of T cells with reduced immune response owing to metabolic dysregulation caused by obesity. This reduced immune response is linked to the decreased functional activation of CD4 and CD8 T cells and the presence of ACE2.^34,35^ ACE, which is also expressed in adipose tissue, acts as a receptor for SARS-CoV-2 in a way that favors the maintenance of the inflammatory state in obese individuals and, consequently, increases the risk of serious outcomes.^35,36,37^ Dysregulated immune responses to the virus tend to compromise other systems that were not previously infected owing to the presence of infected macrophages in the systemic circulation and impaired generation of antibodies, which cause immune suppression in infected obese individuals.^38^

Immune dysregulation, which tends to occur in obese individuals affected by COVID-19, may be related to a phenomenon known as hypercytokinemia or “cytokine storm.”^39^ However, Brandão et al.^40^ have indicated that the initial immune response is very weak considering the high SARS-CoV-2 viral load, which justifies the excessive recruitment of cytokines. This mechanism of action is aggravated by viral overload in epithelial cells, especially in the lungs, causing the collapse of these structures. Thus, hypercytokinemia can occur in individuals infected with SARS-CoV-2 owing to high levels of cytokine expression, mediated by the high initial viral load.^41,42^ Elevated levels of IL-1, IL-2, and IL-6 are detected in severely ill individuals, despite being present since the early stages of COVID-19.^43^ Therefore, the high expression of these interleukins, associated with high concentrations of alpha interferons, beta interferons, and Th1 cells, promotes the constant maintenance of the inflammatory state, resulting in a “cytokine storm.”^44^ Overall, the dysfunctional visceral adipose tissue in obese individuals promotes an inflammatory state after SARS-CoV-2 infection, which impairs their pulmonary structures and, consequently, increases the probability of morbidity and mortality.^45,46^

Another concern with SARS-CoV-2 infection in the obese population is the occurrence of hypercoagulation in the pulmonary structures, which increases the risk of venous thrombosis and pulmonary embolism. Coagulopathies arise from the hyperinflammatory state, especially in lung structures, in obese patients infected with SARS-CoV-2; these are more evident in the more severe stages of COVID-19.^40^ The levels of D-dimer, a blood marker of thrombin levels that is associated with cytokines during hypercytokinemia, are elevated in individuals affected by this coronavirus.^47^ Hypercoagulation is responsible for the change in D-dimer levels, providing insight into the mechanism of COVID-19 worsening in obese individuals. D-dimer levels are further elevated in critically ill hospitalized patients and are associated with worse outcomes in obese individuals.^6,38^ The French Society of Vascular Medicine suggests that obese individuals with COVID-19 are more susceptible to longer hospital stays and intubation times.^48^ This scenario increases the risk of lung injuries and small blood vessel injuries. Thus, given the reduced mobility during hospitalization and hypercoagulability caused by multiple lesions, obese individuals should receive treatment for thromboembolism to avoid the worsening of this disease.^49^

Obstructive sleep apnea syndrome (OSAS) may be a risk factor for the worsening of COVID-19 in obese patients. Strausz et al.^50^ have indicated that there is no difference in the risk of contracting COVID-19 between obese individuals with OSAS and those without the disorder; however, there is a greater risk of the worsening of the disease in obese individuals with OSAS.

Individuals with OSAS have high levels of oxidative stress and are prone to acute respiratory distress syndrome; thus, OSAS is a risk factor for the worsening of COVID-19 in obese patients.^51^ Some studies have shown that the onset of severe lung injuries, such as acute respiratory distress syndrome, after SARS-CoV-2 infection, is a stress coupling mechanism in gravity-dependent and active stress caused by nuclear factor-κB, which provokes an exacerbated pro-inflammatory response under stress.^52,53^ Among the causes for this, repetitive episodes of apnea and the consequent reduction in oxygen saturation stand out. Thus, OSAS can contribute to hospitalization and the use of artificial mechanical ventilation.^54^

**Figure 2.**
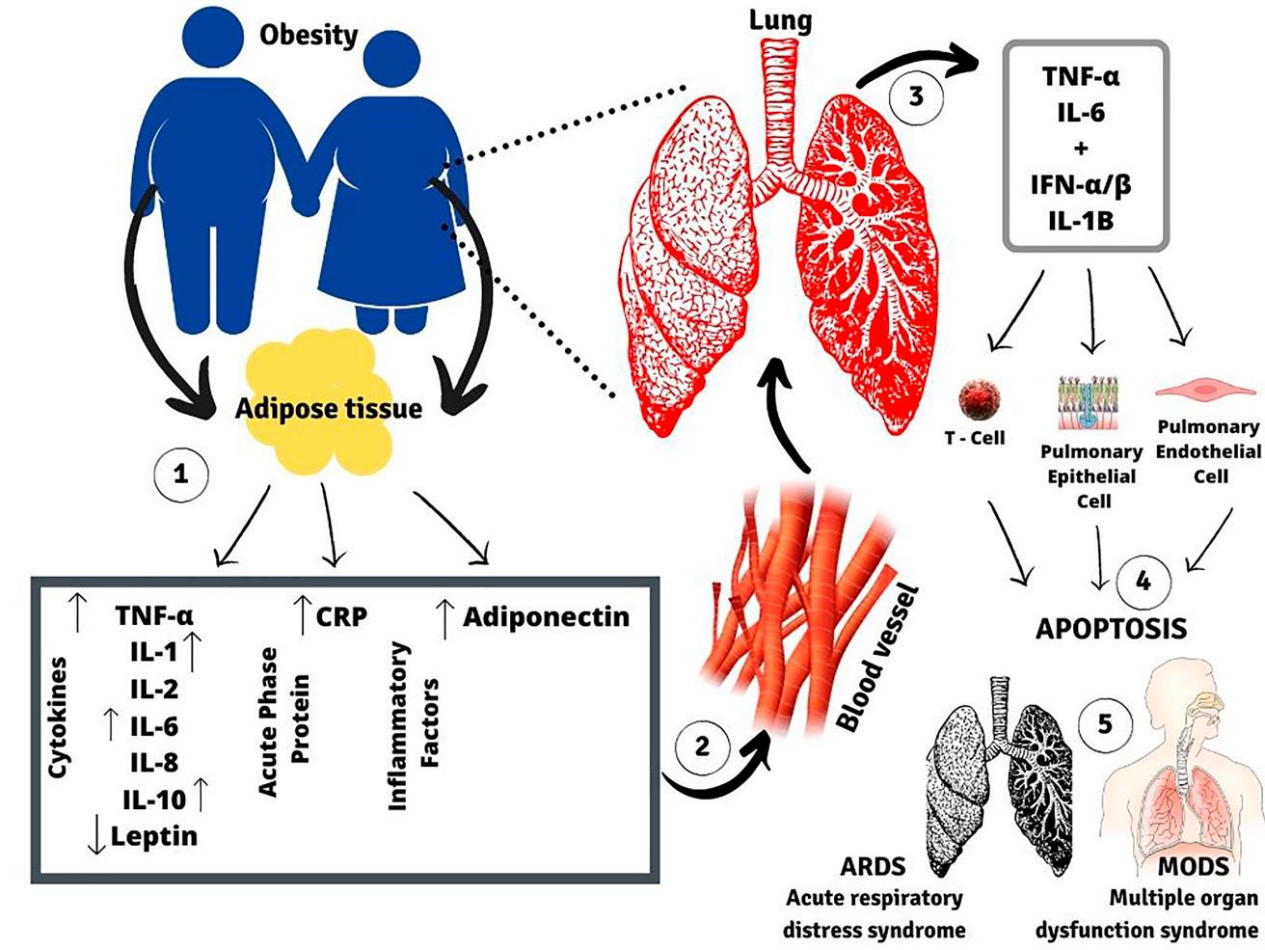
Adipose tissue secretes several pro-inflammatory cytokines, acute phase proteins, and adipokines (1) into the circulation (2), contributing to systemic low-grade chronic inflammation. Obesity-related inflammatory cytokines, in addition to pro-inflammatory molecules related to intrapulmonary (3) SARS-CoV-2, decrease the activation of effector cells in the immune system, which suppresses the immune function and host defenses; in addition, apoptosis of epithelial and pulmonary endothelial cells occurs (4), which can cause lung injuries (5).

Cellular mechanisms contribute to the effects of OSAS in obese patients with coronaviruses. Sleep apnea episodes can cause hypoxemia and, consequently, an inflammatory state, as the low concentration of oxygen in the arterial blood stimulates the release of IL-6 and TNF-α. As a result, the immune response is dysregulated and the respiratory condition worsens, increasing the susceptibility of obese individuals with COVID-19 to acute respiratory distress syndrome.^41^ This indicates the need for a differentiated approach to treatment in obese patients with OSAS affected by this coronavirus, as this population is more susceptible to respiratory failure and, consequently, more severe outcomes (Figure 2).^55,56^

CRP = C-reactive protein; IL = interleukin; TNF-α = tumor necrosis factor-alpha; IFN-α/β = interferons.

### Physical exercise and obesity: Health consequences associated with COVID-19

Physical exercise may be one of the key measures to improve the inflammation in obese individuals with COVID-19.^57^ Regular physical exercise can reduce the number and size of adipocytes, as well as reduce the inflammatory response and associated with immune dysregulation mediated by excess adipose tissue .^58^ A reduction in cytokine expression can be considered a benefit of physical exercise in this population because, among various mechanisms, TNF-α induces an increase in the levels of protein phosphatase 2C; this reduces the activity of AMPK, which is directly linked to the oxidation of fatty acids in muscle and liver tissue and, consequently, control of adiposity.^45^ The entire process of lipid metabolism can benefit from regular physical exercise, which will reduce obesity and the occurrence of problems in individuals affected by this coronavirus.^57^

Physically inactive obese individuals with COVID-19 should participate in regular physical exercise to experience the positive effects of physical exercise on the immune system; the volume and intensity of exercise are crucial factors underlying the beneficial effects of physical exercise in this population.^59^ High intensity (> 75% VO₂ max) and prolonged (> 60 min) physical exercise can result in an immunosuppressive effect, especially in those with systemic impairment, owing to several acute physiological responses, such as micro injuries in target muscles and the consequent increase in the production of ferritin and creatine kinase, which are important markers of muscle inflammation and injury.^60^ Creatine kinase, in particular, is found at greater concentrations in environments with high energy demand, such as muscle tissue, during high-intensity physical exercise.^61^ Mechanical stress caused by excessive muscle contractions, associated with metabolic stress, promotes, in addition to tissue microdamage, an increase in the production of prostaglandins and leukotrienes, accumulation of mitochondrial calcium accumulation, and levels of reactive oxygen species, which cause inflammation.^60,62^ The latter results in the production of receptors for cytokines, such as IL-6 and TNF-α, and exacerbated functional activation of immune system defense cells; for example, the levels of natural killer cells increase 3-fold compared with pre-exercise levels.^63^

Aerobic exercise of low (< 50% VO₂ max) and moderate (≥ 50% and ≤ 75% VO₂ max) intensities lasting up to 60 min does not result in exacerbated inflammatory responses in obese individuals; consequently, the risk of immunosuppression is low, demonstrating the beneficial potential of such exercise in the post-COVID-19 recovery period.^7,45,62^ In obese individuals, regular physical exercise reduces body fat levels, regulates the immune system, and reduces the hyperinflammatory state, resulting in improved outcomes in those with this coronavirus.^18^ Several molecular factors related to the better prognosis of these individuals are mediated by regular physical exercise.^57^

### Angiotensin-2 converting enzyme and adipose tissue

ACE2 is expressed in adipocytes and acts as a receptor for COVID-19 SARS-CoV-2. Its expression tends to decrease in response to regular physical exercise because the size of adipocytes decreases. Considering the high expression of this enzyme in dysfunctional adipose tissue,^37^ the reduction of body and visceral adipose tissue, mediated by physical exercise, may enable a reduction in the risk of infection and degree of inflammation. Regular physical exercise, associated with reductions in lipid profile and dysfunctional adipose tissue as one of the major outcomes, may result in a reduction in the inflammatory condition of this population, considering that excessive visceral adipose tissue in individuals with obesity is related to the release of adipokines and other inflammatory cytokines, such as IL-1, IL-2, IL-6, and alpha and beta interferons.^43^

### Nitric oxide and lung and vascular health

The inflammation of endothelial and vascular dysfunction in individuals with obesity and COVID-19 reduces circulating nitric oxide and increases free radical levels.^2,64^ Free radicals, which are produced owing to oxidative stress in this population, contribute to a worse prognosis in the inflammatory condition and, consequently, a worse clinical respiratory outcome.^64,65^ Regular PA has positive hemodynamic effects, such as increased vasodilation and angiogenesis, mediated by the increased supply of nitric oxide; this is facilitated by the increase in nuclear factor κB levels and consequent increase in the expression of the nitric oxide synthase enzyme in endothelial and neuronal cells caused by physical effort and muscle contraction.^66^ This cascade of events results in the activation of AMPK via the canonical pathway. The progressive increase in the adenosine diphosphate/adenosine triphosphate ratio in the cellular environment mediates the phosphorylation of the AMPK enzyme; this results in gene regulation and the increased expression of the PGC-1α protein, favoring mitochondrial biogenesis and, owing to the translocation of glucose transporter type 4 to the membrane, glycolytic metabolism and lipid oxidation, reducing the lipid and inflammatory profile in individuals with obesity.^67^ Given this mechanism of action of nitric oxide on the vascular endothelium, the maintenance of homeostasis and local blood flow may be promoted.^62^ Thus, regular moderate aerobic exercise may facilitate the recovery of obese individuals from COVID-19.^58^

### PGC-1α and free fatty acids

PGC-1α is a transcriptional coactivator that is involved in the control of several biological mechanisms involved in energy metabolism. For example, a recent study showed the ability of the PGC-1α protein to inhibit the production of IL-6 in hepatocytes, highlighting the possible benefit of this mechanism in clinical conditions involving high levels of inflammation.^68^ Regular moderate-intensity physical exercise tends to increase the expression of PGC-1α because, during physical exercise, the increase in muscle contractions increases the calcium concentration in the sarcoplasm; this results in the activation of calcium-dependent proteins, which alter the phosphorylation state of some transcription factors, such as nuclear factor of activated T cells, and transcription of genes associated with physical exercise, such as PGC-1α.^62,69^ PGC-1α stimulates the production of irisin, a hormone involved in lipid metabolism, especially in adipose tissue, and the immune system, modulating the activity of macrophages.^70,71^ This can reduce the inflammatory picture, increase the enzymatic capacity for free fatty acid oxidation, increase mitochondrial biogenesis, and improve respiratory function.^42,62^

## CONCLUSION

Once exposed to SARS-CoV-2, a hyperinflammatory state and worse prognosis, with a greater risk of hospitalization, are observed in obese individuals. However, regular moderate-intensity PA seems to exert a protective effect against the worsening of health and mortality and is an important tool in the post-infection recovery phase in this population.

## References

[1] Esakandari H, Nabi-Afjadi M, Fakkari-Afjadi J (2020). A comprehensive review of COVID-19 characteristics. Biol Proced Online..

[2] Machhi J, Herskovitz J, Senan AM (2020). The Natural History, Pathobiology, and Clinical Manifestations of SARS-CoV-2 Infections. J Neuroimmune Pharmacol..

[3] Altuntas M, Yilmaz H, Guner AE (2021). Evaluation of patients with COVID-19 diagnosis for chronic diseases. Virol J..

[4] Ho JS, Fernando DI, Chan MY, Sia CH (2020). Obesity in COVID-19: A Systematic Review and Meta-analysis. Ann Acad Med Singap..

[5] Zhu Z, Hasegawa K, Ma B (2020). Association of obesity and its genetic predisposition with the risk of severe COVID-19: analysis of population-based cohort data. Metabolism..

[6] McNeill JN, Lau ES, Paniagua SM (2021). The role of obesity in inflammatory markers in COVID-19 patients. Obes Res Clin Pract..

[7] Jakicic JM, Powell KE, Campbell WW (2019). Physical Activity and the Prevention of Weight Gain in Adults: A Systematic Review. Med Sci Sports Exerc..

[8] OMS (2020). WHO Guidelines on physical activity and sedentary behaviour.

[9] Salman D, Vishnubala D, Le Feuvre P (2021). Returning to physical activity after covid-19. BMJ..

[10] Hudson GM, Sprow K (2020). Promoting physical activity during the COVID-19 pandemic: implications for obesity and chronic disease management. J Phys Act Health..

[11] Diels S, Vanden Berghe W, Van Hul W (2020). Insights into the multifactorial causation of obesity by integrated genetic and epigenetic analysis. Obes Rev..

[12] Herrera BM, Keildson S, Lindgren CM (2011). Genetics and epigenetics of obesity. Maturitas.

[13] Rohde K, Keller M, la Cour Poulsen L (2019). Genetics and epigenetics in obesity. Metabolism.

[14] Gregor MF, Hotamisligil GS (2011). Inflammatory mechanisms in obesity. Annu Rev Immunol.

[15] Ellulu MS, Patimah I, Khaza’ai H, Rahmat A, Abed Y (2017). Obesity and inflammation: the linking mechanism and the complications. Arch Med Sci..

[16] Zeyda M, Stulnig TM (2009). Obesity, inflammation, and insulin resistance--a mini-review. Gerontology..

[17] Zatterale F, Longo M, Naderi J (2020). Chronic Adipose Tissue Inflammation Linking Obesity to Insulin Resistance and Type 2 Diabetes. Front Physiol.

[18] Collao N, Rada I, Francaux M, Deldicque L, Zbinden-Foncea H (2020). Anti-Inflammatory Effect of Exercise Mediated by Toll-Like Receptor Regulation in Innate Immune Cells - A Review. Int Rev Immunol.

[19] Lee YH, Pratley RE (2005). The evolving role of inflammation in obesity and the metabolic syndrome. Curr Diab Rep..

[20] Gralinski LE, Sheahan TP, Morrison TE (2018). Complement Activation Contributes to Severe Acute Respiratory Syndrome Coronavirus Pathogenesis. mBio..

[21] Anty R, Bekri S, Luciani N (2006). The inflammatory C-reactive protein is increased in both liver and adipose tissue in severely obese patients independently from metabolic syndrome, Type 2 diabetes, and NASH. Am J Gastroenterol..

[22] Choi J, Joseph L, Pilote L (2013). Obesity and C-reactive protein in various populations: a systematic review and meta-analysis. Obes Rev..

[23] Dai ZH, Xu XT, Ran ZH (2020). Associations Between Obesity and the Effectiveness of Anti-Tumor Necrosis Factor-α Agents in Inflammatory Bowel Disease Patients: A Literature Review and Meta-analysis. Ann Pharmacother..

[24] Speretta GF, Leite RD, Duarte AC (2014). Obesidade, inflamação e exercício: foco sobre o TNF-alfa e IL-10. Rev Hosp Univ Pedro Ernesto..

[25] Winkler G, Kiss S, Keszthelyi L (2003). Expression of tumor necrosis factor (TNF)-α protein in the subcutaneous and visceral adipose tissue in correlation with adipocyte cell volume, serum TNF-α, soluble serum TNF-receptor-2 concentrations and C-peptide level. Eur J Endocrinol..

[26] Dixon D, Meng H, Goldberg R, Schneiderman N, Delamater A (2009). Stress and body mass index each contributes independently to tumor necrosis factor-α production in prepubescent Latino children. J Pediatr Nurs..

[27] Zorena K, Jachimowicz-Duda O, Ślęzak D, Robakowska M, Mrugacz M (2020). Adipokines and obesity. Potential link to metabolic disorders and chronic complications. Int J Mol Sci..

[28] Stolarczyk E (2017). Adipose tissue inflammation in obesity: a metabolic or immune response?. Curr Opin Pharmacol..

[29] Wood IS, de Heredia FP, Wang B, Trayhurn P (2009). Cellular hypoxia and adipose tissue dysfunction in obesity. Proc Nutr Soc..

[30] Trayhurn P (2013). Hypoxia and adipose tissue function and dysfunction in obesity. Physiol Rev..

[31] Engin A (2017). Adipose tissue hypoxia in obesity and its impact on preadipocytes and macrophages: hypoxia hypothesis. Adv Exp Med Biol..

[32] Graßmann S, Wirsching J, Eichelmann F, Aleksandrova K (2017). Association Between Peripheral Adipokines and Inflammation Markers: A Systematic Review and Meta-Analysis. Obesity (Silver Spring)..

[33] Cai Q, Chen F, Wang T (2020). Obesity and COVID-19 Severity in a Designated Hospital in Shenzhen, China. Diabetes Care..

[34] Stefan N, Birkenfeld AL, Schulze MB (2021). Global pandemics interconnected - obesity, impaired metabolic health and COVID-19. Nat Rev Endocrinol..

[35] Zhou Y, Chi J, Lv W, Wang Y (2021). Obesity and diabetes as high-risk factors for severe coronavirus disease 2019 (Covid-19). Diabetes Metab Res Rev..

[36] Stolarczyk E (2017). Adipose tissue inflammation in obesity: a metabolic or immune response?. Curr Opin Pharmacol.

[37] Al Heialy S, Hachim MY, Senok A (2020). Regulation of Angiotensin- Converting Enzyme 2 in Obesity: Implications for COVID-19. Front Physiol..

[38] Mostaghim A, Sinha P, Bielick C (2020). Clinical outcomes and inflammatory marker levels in patients with Covid-19 and obesity at an inner-city safety net hospital. PLoS One..

[39] Mehta P, McAuley DF, Brown M (2020). COVID-19: consider cytokine storm syndromes and immunosuppression. Lancet..

[40] Brandão SCS, Godoi ETAM, de Oliveira Cordeiro LH (2021). COVID-19 and obesity: the meeting of two pandemics. Arch Endocrinol Metab..

[41] Ackermann M, Verleden SE, Kuehnel M (2020). Pulmonary Vascular Endothelialitis, Thrombosis, and Angiogenesis in Covid-19. N Engl J Med..

[42] Ayres JS (2020). A metabolic handbook for the COVID-19 pandemic. Nat Metab..

[43] Chowdhury MA, Hossain N, Kashem MA, Shahid MA, Alam A (2020). Immune response in COVID-19: A review. J Infect Public Health..

[44] Kimura T, Namkoong H (2020). Susceptibility of the obese population to COVID-19. Int J Infect Dis..

[45] Luzi L, Radaelli MG (2020). Influenza and obesity: its odd relationship and the lessons for COVID-19 pandemic. Acta Diabetol..

[46] de Siqueira JV, Almeida LG, Zica BO (2020). Impact of obesity on hospitalizations and mortality, due to COVID-19: A systematic review. Obes Res Clin Pract..

[47] Gayam V, Chobufo MD, Merghani MA (2021). Clinical characteristics and predictors of mortality in African-Americans with COVID-19 from an inner-city community teaching hospital in New York. J Med Virol..

[48] Khider L, Soudet S, Laneelle D (2020). French Society of Vascular Medicine (SFMV). Proposal of the French Society of Vascular Medicine for the prevention, diagnosis, and treatment of venous thromboembolic disease in outpatients with COVID-19. J Med Vasc..

[49] Ullah W, Saeed R, Sarwar U, Patel R, Fischman DL (2020). COVID-19 Complicated by Acute Pulmonary Embolism and Right-Sided Heart Failure. JACC Case Rep..

[50] Strausz S, Kiiskinen T, Broberg M (2021). FinnGen: sleep apnoea is a risk factor for severe COVID-19. BMJ Open Respir Res..

[51] Delgado-Roche L, Mesta F (2020). Oxidative Stress as Key Player in Severe Acute Respiratory Syndrome Coronavirus (SARS-CoV) Infection. Arch Med Res..

[52] Padhan K, Minakshi R, Towheed MAB, Jameel S (2008). Severe acute respiratory syndrome coronavirus 3a protein activates the mitochondrial death pathway through p38 MAP kinase activation. J Gen Virol..

[53] Smith JT, Willey NJ, Hancock JT (2012). Low dose ionizing radiation produces too few reactive oxygen species to directly affect antioxidant concentrations in cells. Biol Lett..

[54] Chu Y, Yang J, Shi J, Zhang P, Wang X (2020). Obesity is associated with increased severity of disease in COVID-19 pneumonia: a systematic review and meta-analysis. Eur J Med Res..

[55] de Kruif MD, Voncken SFJ, Laven SAJS, Feron TMH, Kolfoort-Otte AAB (2021). Obstructive sleep apnea and risk of COVID-19 infection, hospitalization, and respiratory failure. Sleep Breath..

[56] Miller MA, Cappuccio FP (2021). A systematic review of COVID-19 and obstructive sleep apnoea. Sleep Med Rev..

[57] da Silveira MP, da Silva Fagundes KK, Bizuti MR (2021). Physical exercise as a tool to help the immune system against COVID-19: an integrative review of the current literature. Clin Exp Med..

[58] Fernández-Lázaro D, González-Bernal JJ, Sánchez-Serrano N (2020). Physical exercise as a multimodal tool for COVID-19: could it be used as a preventive strategy?. Int J Environ Res Public Health..

[59] Leandro CG, Ferreira E, Silva WT, Lima-Silva AE, Lima-Silva AE, Lima-Silva AE (2020). Covid-19 and Exercise-Induced Immunomodulation. Neuroimmunomodulation..

[60] Baird MF, Graham SM, Baker JS, Bickerstaff GF (2012). Creatine-kinase- and exercise-related muscle damage implications for muscle performance and recovery. J Nutr Metab..

[61] Ertel KA, Hallam JE, Hillman AR (2020). The effects of training status and exercise intensity on exercise-induced muscle damage. J Sports Med Phys Fitness..

[62] Nigro E, Polito R, Alfieri A (2020). Molecular mechanisms involved in the positive effects of physical activity on coping with COVID-19. Eur J Appl Physiol..

[63] Furtado GE, Letieri RV, Caldo-Silva A (2021). Sustaining efficient immune functions with regular PE in the COVID-19 era and beyond. Eur J Clin Invest..

[64] Lisi F, Zelikin AN, Chandrawati R (2021). Nitric Oxide to Fight Viral Infections. Adv Sci.

[65] Virdis A, Colucci R, Bernardini N (2018). Microvascular Endothelial Dysfunction in Human Obesity: Role of TNF-α. J Clin Endocrinol Metab..

[66] Silveira EM, Rodrigues MF, Krause MS (2007). Acute exercise stimulates macrophage function: possible role of NF-kappaB pathways. Cell Biochem Funct..

[67] Dyakova EY, Kapilevich LV, Shylko VG, Popov SV, Anfinogenova Y (2015). PE associated with NO production: signaling pathways and significance in health and disease. Front Cell Dev Biol..

[68] Barroso WA (2016). PGC-1 alfa como regulador inflamatório na esteato-hepatite não-alcoólica [thesis].

[69] Liang H, Ward WF (2006). PGC-1α: a key regulator of energy metabolism. Adv Physiol Educ..

[70] Korta P, Pocheć E, Mazur-Biały A (2019). Irisin as a multifunctional protein: implications for health and certain diseases. Medicina (Kaunas)..

[71] Blizzard LeBlanc DR, Rioux BV, Pelech C (2017). Exercise-induced irisin release as a determinant of the metabolic response to exercise training in obese youth: the EXIT trial. Physiol Rep..

